# Nutraceutical Oils Produced by Olives and *Citrus* Peel of Tuscany Varieties as Sources of Functional Ingredients

**DOI:** 10.3390/molecules24010065

**Published:** 2018-12-25

**Authors:** Roberta Ascrizzi, Isabella Taglieri, Cristina Sgherri, Guido Flamini, Monica Macaluso, Chiara Sanmartin, Francesca Venturi, Mike Frank Quartacci, Luisa Pistelli, Angela Zinnai

**Affiliations:** 1 Department of Pharmacy, University of Pisa, Via Bonanno Pisano 6, 56126 Pisa, Italy; roberta.ascrizzi@gmail.com (R.A.); guido.flamini@unipi.it (G.F.); luisa.pistelli@unipi.it (L.P.); 2 Department of Agriculture, Food and Environment, University of Pisa, Via del Borghetto 80, 56124 Pisa, Italy; isabella.taglieri@for.unipi.it (I.T.); cristina.sgherri@unipi.it (C.S.); francesca.venturi@unipi.it (F.V.); mike.frank.quartacci@unipi.it (M.F.Q.); angela.zinnai@unipi.it (A.Z.); 3 Interdepartmental Research Center, Nutraceuticals and Food for Health, University of Pisa, Via del Borghetto 80, 56124 Pisa, Italy

**Keywords:** SPME, gas chromatography-mass spectrometry, essential oils, volatile organic compounds, *Citrus*, carotenoids, naringenin, tyrosol, hydroxytyrosol

## Abstract

The essential oils extracted from the peels of two Tuscany *Citrus* of the Massa province have been characterised. Moreover, the flavedo of these species has been used in the production of two *Citrus* olive oils (*C*OOs) obtained with an innovative method in which the citrus peels are cryomacerated and then pressed with the olives. The presence of functional compounds, such as carotenoids, naringenin and minor phenolics, classifies these *C*OOs as nutraceuticals with the potential to develop enriched foods able to promote a healthy diet. Moreover, the increased presence of tyrosol and hydroxytyrosol, compared to the unflavoured oil, further highlights the nutritional value to the two *C*OOs, being these phenolic compounds recognized as good possible therapeutic candidates for the inhibition of neurodegenerative diseases as the Parkinson’s disease. In this perspective, the citrus peels, rich in bioactive compounds, have been valued transforming their waste nature in an innovative resource.

## 1. Introduction

*Citrus* tree cultivation has a long history in the Massa-Carrara province (Tuscany, Italy) [1,2,3] and, thanks to the climate of this area, whose position ensures protection from the wind and mild temperatures due to the sea proximity, its *Citrus* fruits reach peculiar attributes, distinguishing them from the Southern varieties [2]. Lemons (*C. limon* (L.) Osbeck) exhibit a round shape and medium size [2,4,5]. Their main features are the fine peel and the bittersweet flavour, for which they can also be consumed fresh; furthermore, they show a longer post-harvest life, with low to absent rotting [2,4,5]. Oranges (*C. sinensis* (L.) Osbeck) show a round-shaped morphology, with a light orange colour in both the peel and the pulp, and a medium-to-small size [1,6]. Like the lemons from this area, their peels are thin: they are used in the confectionery sector to produce the candied peels [1,6]. The pulp is rich in juice, characterised by a bittersweet flavour and pleasant smell [1,6].

A strategic line for the *Citrus* agro-food sector could be the recovery of its by-products, namely peels and seeds, to be used in the formulation of innovative foods able to combine nutritional and health properties and therefore to meet the changing needs of the consumers. This could ensure the exploitation of all the parts of the fruit, without any waste. In the last few years, the use of by-products of the food industry as natural sources of bioactive compounds has been widely considered, and several studies focused on the development of innovative products [7,8]. The enhancement of agricultural by-products could realise a circular economy model, as well as become a driving force for the development of marginal territories. Extra-virgin olive oil (EVOO) is a commodity which enjoys particular prestige in the globalized economy and Tuscany contributes also to the Italian production of EVOO with ideal, typical-high quality products, symbolic of consumer trends. “Frantoio” and “Leccino” are famous Tuscan-blend oils with characteristic strong, aromatic, grassy, fruity flavor, strong pungency and with its own spicy character. For these reasons, Tuscan oils have considerable market recognition all over the world. Increased consumption of olive oil, in place of animal-derived fats, has been associated with a reduced incidence of age-associated diseases, including cardiovascular diseases and cancer, as well as neurodegenerative diseases [8], due to the presence in olive oil of bioactive compounds such as polyphenols. Moreover, the development of new extraction methods based on the production of functional foods enriched with natural antioxidants has been demonstrated to be promising as potential application for the stabilization of olive oil and the increase of its shelf life [8]. Thus, a novel food characterized by enhanced properties, able to combine the expectations of the modern consumer with the promotion of local products, could represent the winning key of this sector.

The aim of this research was to produce and characterize, from a chemical and sensory point of view, two citrus-flavoured olive oils (*C*OOs) in comparison with an unflavoured EVOO. *C*OOs were obtained through the direct addition of cryo-macerated citrus peels (from lemon and orange of Massa Carrara province, Tuscany) in the olive mill, together with fresh olives (from Tuscany varieties too) during oil extraction [9,10]. Since citrus peel is rich in polyphenols [11] and characterized by the presence of typical essential oils [12], analyses of the main phenolic compounds as well as of the volatile organic compounds in *C*OOs were performed. Essential oils of the citrus peels added for *C*OOs production were also investigated.

In particular, the attention was focused on the possibility to obtain typical products, with additional bioactive compounds, through the recovery of the by-products of *Citrus* fruits (peels of lemon and orange) which, containing high levels of polyphenols, have attracted scientific interest due to their potent antimicrobial and antiradical activities [11].

## 2. Results and Discussion

### 2.1. Chemical Composition of the Essential Oils (EOs)

#### 2.1.1. Lemon Essential Oils

The complete compositions of the EOs and the extraction yields of the lemon peels are reported in Table 1. Six compounds were identified in the volatile fraction obtained by manual squeezing of the lemon flavedo, all belonging to the monoterpene hydrocarbon chemical class. Limonene represented over 70% of the total composition, followed by β-pinene (14.9%) and γ-terpinene (9.6%). All the compounds found in this volatile fraction were also detected in the flavedo hydrodistilled EO, in which 33 compounds were identified. The hydrodistilled lemon flavedo EO was dominated (over 75%) by monoterpene hydrocarbons, and the most abundant compounds were the same of the manually squeezed volatile fraction: limonene (50.2%), β-pinene (10.6%) and γ-terpinene (9.6%). Oxygenated monoterpenes accounted for 20.1%: geranial (7.1%) and neral (5.7%) showed the highest relative abundances, and their correlated alcohols were detected as well, but in lower relative quantities (0.8%). Only 3 compounds were exclusively found in this EO among all those extracted from the lemon samples, but all in a relative abundance lower than 0.5%. 

As reported in published lemon peel EO compositions, limonene is the most relevant compound, followed by β-pinene and γ-terpinene among the monoterpene hydrocarbons, and by neral and geranial among the oxygenated ones [12]. 

#### 2.1.2. Orange Essential Oils

The complete composition of the EOs and the extraction yields of the orange samples is reported in Table 2. The manually squeezed orange volatile fraction was dominated by monoterpene hydrocarbons (over 98%), among which limonene was the most abundant (91.4%), followed by sabinene (3.2%) and myrcene (2.6%). Among the 10 compounds detected in this EO, only octanaland (E)-β-ocimene were not shared with the flavedo hydrodistilled EO, characterized by the presence of 17 different compounds. The hydrodistilled orange flavedo EO was dominated (over 90%) by monoterpene hydrocarbons, and the most abundant compound was the same of the manually squeezed volatile fraction: limonene (85.7%). Other compounds present at significant amounts were represented by sabinene (1.9%), myrcene (2.2%), octanal (2.0%) and linalool (3.5%). In contrast with lemon, oxygenated monoterpenes of orange EO accounted only for 5.6% in favour of an increased presence of limonene.

### 2.2. The Citrus Olive Oils (COOs)

#### 2.2.1. Volatiles Bouquet in the Headspace Emissions of the *C*OOs

The complete composition of headspace volatile emissions of the *C*OOs and of the unflavoured olive oil control sample is reported in Table 3. The spontaneous volatile emission of the *C*OOs was drastically altered compared to the control. The latter, indeed, mainly emitted non-terpene compounds: they accounted for up to 92.0% of its total headspace emission, of which over 80% was represented by (*E*)-2-hexenal. The relative abundance of the latter decreased almost to trace amounts in the flavoured samples, which exhibited volatile emissions dominated by monoterpene hydrocarbons: 99.1% for the *Cl*OO (*Citrus limon* olive oil), 98.3% for the *Cs*OO (*Citrus sinensis* olive oil). The most relevant contributions to these headspaces retraced the composition of the manually squeezed volatile fraction of the citrus peels. Limonene accounted for more than 65% of the *Cl*OO headspace, followed by β-pinene (13.6%) and γ-terpinene (8.1%). For the *Cs*OO, limonene dominated the volatile emission, with a relative abundance >90%; myrcene and sabinene followed, accounting for 3.0 and 1.5%, respectively. 

The persistence of the same compounds detected in the manually squeezed volatile fractions of the citrus peels confirmed the capacity of the cryomaceration technology to retain the volatile organic compounds (VOCs) in the final product. It avoided the thermal degradation of the VOCs, which were released in the oil: the lipophilic nature of the latter trapped the aromatic notes, which conferred a more complex bouquet to the final product. The unflavoured olive oil headspace was dominated by the sweet and fruity note [13] of (*E*)-2-hexenal. The *Cl*OO exhibited an aromatic volatile emission characterized by the citrus-like, pleasant limonene notes [13], followed by the woody, resinous and more bitter notes of β-pinene and γ-terpinene [13]. The orange *Cs*OO, instead, showed a sweeter profile, with limonene as the dominant note, followed by the sweet and balsamic notes of myrcene [13]. 

### 2.3. Chemical Characterization of the Citrus Olive Oil 

#### 2.3.1. Quality Parameters

The *C*OOs obtained applying the optimised working parameters for the production of the olive oil, as reported in Section 3.2, were analysed and compared with the reference used by the EU for the determination of the quality of extra-virgin olive oil.

In particular, as reported in Table 4, both *C*OOs showed chemical parameters within the limits established for EVOO by the Regulation EEC/2568/91 and later modifications [14]. Moreover, it is interesting to note that the addition of different citrus by-products to olives during the extraction process did not affect the quality of olive oil (Table 4).

#### 2.3.2. Phenolic Content, Intensity of Bitterness and Antioxidant Capacity

The phenolic compounds exhibit a wide range of biological functions, which have been attributed to their free radical scavenging and antioxidant activities. For all the extracted oils (control EVOO and both *C*OOs), the total phenolic contents are reported in Table 5. While both the *C*OOs showed a reduced amount of total phenols if compared with control EVOO, they still showed a significant antioxidant activity, combined with a lower Intensity of Bitter. These differences in phenolic profiles can be probably due to the interactions among part of astringent phenolic compounds present in olives with citrus peels during the extraction process. [15]. Furthermore, while both *C*OOs showed similar antioxidant capacity values, concerning the total carotenoid content the results were very different between the two *C*OOs, as the orange one exhibited an almost six time higher value than the lemon *C*OO. In fact, the carotenoids are responsible for the external and internal colouration of orange fruits [16], and a large proportion of them was found in the flavedo of the fruits [17] as well. The use of cryomaceration could have favoured the extraction of these compounds, causing their relevant presence in the orange *C*OO. The increase in carotenoid content could be considered a very interesting feature, because the serum levels of these compounds are inversely associated with an impaired glucose metabolism: the lower the carotenoids, the worst the glucose intolerance [18]. Thus, the *C*OOs, rich in carotenoids, could represent an important innovative product, as humans are not capable of synthesizing carotenoids which should be exclusively of dietary derivation. Moreover, there is evidence that dietary intake of carotenoids is linked with reduced risks of several chronic health disorders, including heart disease, age-related macular deterioration and certain cancers [19], and their actions are related to the capacity of carotenoids to quench reactive oxygen species [19].

#### 2.3.3. Flavonoid Composition

The presence of flavonoids in the olive oil control accounted for 5% of the phenolic content (Table 6), a percentage 1.6-fold higher in comparison with that of phenolic acids (Table 7). Since flavonoids are important natural compounds with different biological activities, among which the antioxidative one, their presence deserves particular attention also in the study of the phenolic profile of EVOO. Flavonoid amounts are affected by many factors such as cultivar, ripening stage and orchard [20]; luteolin was one of the main flavonoids detected in the *C*OO extract (Table 6), according to literature [20,21]. The other main flavonoid was kaempferol, which accounted for 39% of the flavonoid content, whereas luteolin-7-*O*-glucoside, rutin, quercetin-3-*O*-glucoside, apigenin-7-*O*-glucoside, quercitrin, quercetin-3-*O*-glucuronide and quercetin were the less represented (Table 6). The extraction procedure differently affected flavonoid composition of control and *C*OOs. Luteolin was detected in high amounts also in *Cl*OO more enriched with luteolin-7-*O*-glucoside and lutein (Table 6). Notwithstanding a general decrease was observed in the other flavonoid compounds, the flavonoid profile of *C*OOs was characterized by the presence of naringenin (26 and 22% of flavonoid content in *Cl*OO and *Cs*OO, respectively). This flavanone, originated from citrus peel addition [22], adds value to *C*OOs. In fact, naringin and its aglycone naringenin belong to citrus flavonoids, which displayed strong anti-inflammatory and antioxidant activities. An increasing amount of research suggests that naringin supplementation is beneficial for the treatment of obesity, diabetes, hypertension, and metabolic syndrome [23]. Moreover, naringin and naringenin exhibited an important role in the oxidative stress protection, being both strong scavengers of free radicals and preventing lipid peroxidation [24,25,26]. Thus, the presence of naringenin can endow *C*OOs with healthy properties as well as preserve them from lipid degradation, increasing their shelf-life.

#### 2.3.4. Composition of Phenolic Alcohols, Aldehydes and Acids

The study of phenolic acid composition, as well as that of the main phenolic alcohols and aldehydes characterising the profile of EVOO, is reported in Table 7. Both *C*OOs (lemon and orange) showed values 3-fold higher in the total content of the phenolics investigated in comparison with the EVOO control. More and more interest in phenolic acids arises from their potential protective role, through ingestion of fruit and vegetables, against diseases that may be related to oxidative damage (coronary heart disease, stroke, and cancers) [27]. Besides the health-related and antioxidant properties of foods, phenolic acids have been associated with color and sensory qualities [28]. Moreover, these compounds can be considered as potential markers of geographical origin or the olive fruit variety.

Composition of the two *C*OOs was quite similar (Table 7). Both of them showed a slight increase in the level of hydroxtyrosol and a 3-fold increase in that of tyrosol. Considerable amounts of hydroxytyrosol and tyrosol have been found in melon peels [29], whereas bitter orange juice contained tyrosol [30]. The more and more recognized antioxidant activity of hydroxytyrosol and tyrosol [8] has sparked research in different fields, the most important being the prevention of cardiovascular diseases [31]. But properties including antitumoral, antimicrobial, antidiabetic, and neuroprotective activities have been also attributed to these minor compounds [32]. Moreover, the thorough analysis of the pharmacokinetic properties and safety profile, as well as the in vitro and in vivo researches of their neuroprotective actions, suggest a role as possible therapeutic candidates for the inhibition of neurodegenerative diseases among which the Parkinson’s disease [33].

Huge increases in vanillic, *p*-coumaric and ferulic acids contents, although more consistent in *Cl*OO, could be attributed to citrus peel addition, in agreement with what found in literature about bitter orange [29] where *p*-coumaric and ferulic acids were the most abundant phenolic compounds representing the 24.7% and 23.8%, respectively, of the peel extract. Bocco et al. [34] reported similar results showing the prevalence of phenolic acids in *Citrus* fruits. Since rosmarinic acid was found in the peel and the juice of bitter orange [30], its presence in *C*OOs can be originated by citrus peel addition too. A wide spectrum of beneficial activity for human health has been advocated for these phenolic compounds, at least in part, because of their strong antioxidant activity. In particular, Zang et al. [35] showed that *p*-coumaric acid can act as a direct scavenger of reactive oxygen species to prevent lipid peroxidation, whereas Kanski et al. [36] highlighted that the presence of electron donating groups on the benzene ring (3 methoxy and more importantly 4-hydroxyl) of ferulic acid gives to this compound the additional property of terminating free radical chain reactions.

Thus, the employ of citrus peel allows to obtain healthy-*C*OOs, richer in phenolic compounds, being *Citrus* fruits rich in phenolics with high antioxidant activity [37].

#### 2.3.5. Sensory Analysis

**Figure 1:**
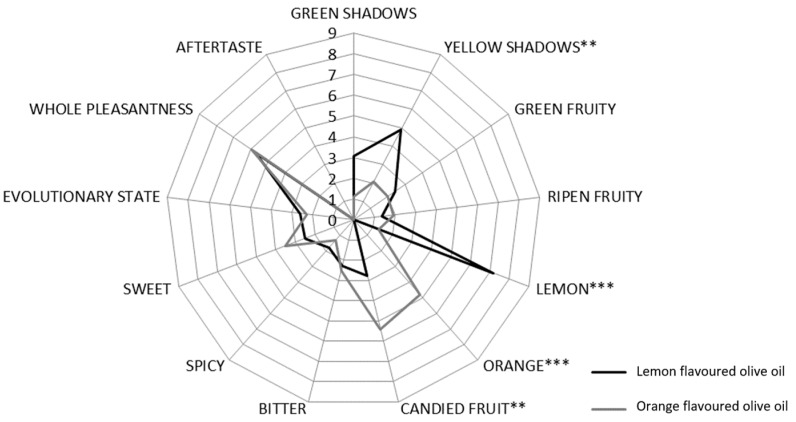
Sensory profile of *Citrus* Olive Oils. Stars meant differences statistically significant (***: *p* < 0.001; **: *p* < 0.01).

Regarding to the sensory profiles of the two citrus olive oils (Figure 1), orange olive oil, characterized by the highest content of carotenoids, was evaluated by the panel as the sample with the highest value for orange shadows of colour.

Both the oils exhibited a quite good intensity of aroma, even if with different scents, as expected considering the typical features of the fruits (Figure 1). A peculiar scent recognized in both samples was the candied fruit, which was higher in the orange flavoured olive oil. 

The taste parameters were globally evaluated as similar by the judges. It is interesting to note that both samples reached values related to bitter and spicy quite low in comparison with a generic olive oil. This issue, together with the olfactory profiles, is very interesting: indeed, both the *C*OOs could be considered not only as dressing for salty food, but also an interesting ingredient for pastry and baking application, due to their sweet note.

Moreover, as expected considering the chemical quality of *C*OOs reported in Table 4, both samples (lemon and orange) showed a good frankness and no panellist perceived any negative attributes (defects); this data seem to be very important for the definition of their merceological classes, according to the legal limits for extra-virgin olive oil provided by the regulation EEC/2568/91 and later modifications and integrations [14].

## 3. Materials and Methods 

### 3.1. Plant Material

*Citrus* samples were produced by a local, individual producer (Aldo Fiorentini) of the Massa province. The flavoured extra-virgin olive oils and the control (not flavoured) were produced from *Frantoio*, *Moraiolo*, and *Leccino* olive varieties provided by a private company located in Tuscany (San Miniato, Pisa, Italy) during the 2017/2018 crop season (Table 8).

### 3.2.Phytochemical Analyses

#### 3.2.1. Essential Oils (EOs) Hydrodistillation

Peel hydrodistillations were performed on fresh material in a Clevenger apparatus equipped with an electric mantle heater for 2 h. Each extraction was performed in triplicate. For the peels, after the albedo removal, the flavedo was roughly cut and hydrodistilled. After the removal of the branches, leaves were roughly cut and hydrodistilled. Furthermore, 5.0 g for each peel sample were manually squeezed in a glass vial containing 1 mL of HPLC grade *n*-hexane. Immediately after each distillation, 1 μL of essential oil was injected after dilution in *n*-hexane HPLC grade at 5% for each replicate.

#### 3.2.2. Headspace Solid Phase Micro-Extraction (SPME) of the *Citrus* Olive Oils (*C*OOs)

The headspace spontaneous volatile emissions of the two citrus-flavoured olive oils were compared to that of the unflavoured control sample. Triplicates were performed for each sample. For each replicate, 2 mL were put in a glass vial closed with aluminium foil. The equilibration was performed at room temperature for 30 min for all the samples before sampling. Solid Phase Micro-Extraction (SPME, Supelco, St. Louis, MO, USA) devices coated with polydimethylsiloxane (PDMS, 100 μm) were used to sampling the headspace of the samples. SPME sampling was performed using the same new fibre, preconditioned according to the manufacturer instructions, for all the analyses. Sampling was accomplished in an air-conditioned room (22 ± 1 °C) to guarantee a stable temperature; sampling time was 3’ for each sample. Once sampling was finished, the fibre was withdrawn into the needle and transferred to the injection port of the GC-MS system. The desorption conditions were identical for all the samples (Section 2.2.1). Furthermore, blanks were performed before each first SPME extraction, and randomly repeated during each series. Quantitative comparisons of relative peaks areas were performed between the same chemicals in the different samples.

#### 3.2.3. Gas Chromatography-Mass Spectrometry Analyses and Peak Identification

The GC/EI-MS analyses were performed with a CP-3800 apparatus (Varian Inc., Palo Alto, CA, USA) equipped with a DB-5 capillary column (30 m × 0.25 mm i.d., film thickness 0.25 μm) and a Varian Saturn 2000 ion-trap mass detector (Varian Inc., Palo Alto, CA, USA). The oven temperature was programmed rising from 60 °C to 240 °C at 3 °C/min; injector temperature, 220 °C; transfer-line temperature, 240 °C; carrier gas, He (1 mL/min). The acquisition parameters were as follows: full scan; scan range: 35–300 *m*/*z*; scan time: 1.0 s; threshold: 1 count. The identification of the constituents was based on the comparison of their retention times (*t*_R_) with those of pure reference samples and their linear retention indices (LRIs) determined relatively to the *t*_R_ of a series of *n*-alkanes. The mass spectra were compared to those listed in the commercial libraries NIST 14 and ADAMS and in a homemade mass-spectral library built up from pure substances and components of known oils, and MS literature data [39,40,41,42,43,44].

### 3.3. Citrus Olive Oil Extraction

Citrus peels were cryomacerated with solid carbon dioxide (1:1 in weight) overnight and then directly added (22% in weight) to olives before milling. The extraction was carried out using a micro oil mill (Oliomio Baby®, produced by “Toscana Enologica Mori”, Tavernelle Val Di Pesa, Florence, Italy) able to mill 20–30 kg of olives. The technical characteristics of the micro oil mill and the working conditions used followed the method previously described [45].

### 3.4. COOs Chemical Analyses

#### 3.4.1. Quality Parameters

Free fatty acids (FFA), peroxide value (PV) and spectrophotometric indices (K232, K270 and ∆K) were determined according to the Official EU analytical methods described in the Regulation EEC/2568/91 and later modifications [14].

#### 3.4.2. Analysis of the Phenolic Content

Total phenols were extracted from the flavoured oil as previously described [46,47] and extracts were stored at −20°C under N_2_ atmosphere until use. The determination of the total phenols was performed according to the Folin-Ciocalteau colorimetric method, using gallic acid as standard.

#### 3.4.3. Antioxidant Capacity Assay

The antioxidant capacity of the phenolic extracts from the flavoured oil samples was performed following the method previously described [48], using the radical cation ABTS (2,2′-azino-bis(3-ethylbenzothiazoline-6-sulphonic acid). The radical solution was prepared as previously described [49], and a Trolox dose-response curve in the 0.2–1.5 mM range was used. The antioxidant activity was expressed as Trolox equivalent antioxidant capacity (TEAC) per mL of extract.

#### 3.4.4. Analysis of Flavonoid Contents 

All reagents were of the highest purity and were purchased from Sigma-Aldrich (Milan, Italy). Water was of Milli Q grade. All solvents and water were accurately degassed before use in the analyses. Analysis of flavonoids was performed by RP-HPLC (Waters S.p.A, Sesto San Giovanni, Milan, Italy). Twenty microliters of phenolic extract were injected into a model 515 HPLC system (Waters S.p.A, Sesto San Giovanni, Milan, Italy) fitted with a 4.6 mm × 250 mm Prodigy ODS column (Phenomenex, Bologna, Italy). A Waters 2487 dual λ UV-visible detector was set at 360 nm. Chromatogram analysis was performed as previously described [50]. Identification of the free flavonoids was performed by co-chromatography on HPLC with authentic standards. Quantification was achieved using standard curves in the range of 10–200 ng of a standard mixture containing luteolin-7-*O*-glucoside, rutin, quercetin-3-*O*-glucoside, apigenin-7-*O*-glucoside, quercitrin, quercetin-3-*O*-glucuronide, quercetin, luteolin, kaempferol and naringenin. Chromatogram analysis was performed by the Millennium 32 software (Version 3.05.01, Waters S.p.A, Sesto San Giovanni, Milan, Italy). 

#### 3.4.5. Analysis of Phenolic Alcohols, Aldehyde ad Acids 

Qualitative and quantitative analyses were performed by RP-HPLC at the chromatographic conditions previously reported [50]. The identity of the phenolic alcohols, aldehyde as well as free phenolic acids was confirmed by chromatography with authentic standards, and quantification was performed using a standard curve in the range 0.1−0.5 μg of standard mixtures containing hydroxytyrosol, tyrosol, vanillin, gallic, protocatechuic, *p*-hydroxybenzoic, chlorogenic, vanillic, caffeic, syringic, *p*-coumaric, ferulic, and rosmarinic acids. Chromatogram analysis was performed as previously reported for flavonoid determination.

#### 3.4.6. Intensity of Bitterness (IB) Determination

The IB was determined following the method previously described [51]. Bitter components were extracted from 1.00 ± 0.01 g *C*OOs samples and octadecyl (C18) disposable extraction columns (6 mL) (J.T. Baker Chemical Company, Phillipsburg, NJ, USA) were used. Absorbance was recorded at 225 nm.

#### 3.4.7. Carotenoids

Carotenoids were determined colorimetrically, at 470 nm, according to the method previously described [52]. This methodology evaluates carotenoid content, an added attribute for estimate olive oil quality. 

#### 3.4.8. Sensory Analysis

The quantitative descriptive analysis of the *C*OOs samples was performed by a panel of 10 trained assessors included in the “expert panel” of the Department of Agriculture, Food and Environment (DAFE) of the University of Pisa, according to the internal procedure for assessor selection and training [53]. 

**Figure 2:**
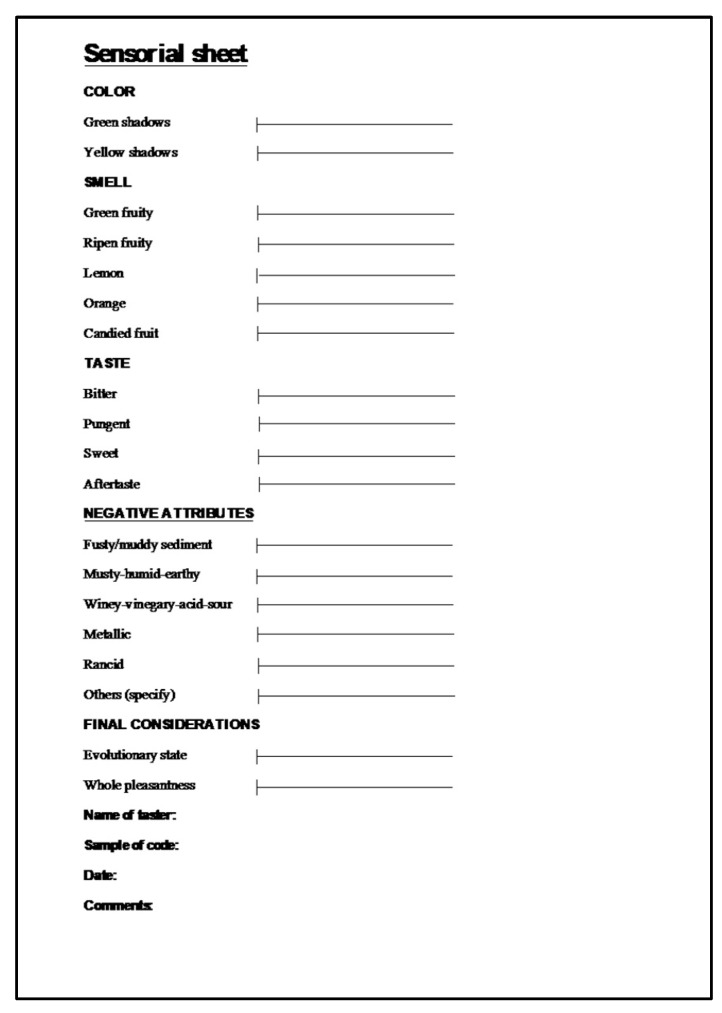
Technical evaluation sheet used for the sensory analysis.

The sensorial characterisation followed the method described in the EEC/2568/91 Regulation and later modifications [14]. To better describe the organoleptic evolution of the flavoured oil samples, the panel was provided with a technical evaluation sheet, specifically developed for this purpose (Figure 2).

Using the proposed sheet, it was possible to obtain a sensory profile of the *C*OOs on the basis of (i) the first order descriptors of colour, flavouring and taste and of (ii) the hedonic parameter related to the evolutionary state and overall pleasantness. Particular attention was paid to the perception of some smell parameters related to citrus fruits (i.e., lemon, orange, candied fruit). Differently from the International Olive Council [54] guidelines, which provide for the use of a dark-coloured glass, we used a transparent glass in order to better evaluate the colour of the *C*OOs as the peel of the citrus fruit could differently influence the final colour of the final product. The panellists ranked the flavoured oil samples on a scale from 0 (no perception, the lowest intensity) to 9 (the highest intensity) to evaluate the intensity of each parameter. The tasting was carried out in the conditions previously described [55].

#### 3.4.9. Statistical Analysis

The results are the means ± SD of three independent experiments. The significance of differences among means was determined by one-way ANOVA (CoStat, Version 6.451, CoHort Software, Pacific Grove, CA, USA). Comparisons among means were performed by the Bartlett’s X2 corrected test (*p* < 0.05). 

## 4. Conclusions

Citrus fruit by-products, such as peels, can be further exploited for the extraction of essential oils and for the aromatization of olive oils with the result of typical, added-value products. In fact, the use of cryomacerated *Citrus* peels as flavouring agents in the olive oil extraction revealed the ability of this technology to retain the typical aroma notes of these plants in the final product, which showed a complex sensory profile. The technology adopted in the production of *C*OOs showed to be also able to enrich oils with functional and bioactive compounds, mostly from citrus peel. Examples are carotenoids, naringenin, tyrosol and hydroxytyrosol, whose health benefits are more and more recognised. In particular, naringenin, abundantly found in citrus fruit, adds value to the *C*OOs, having recently drawn scientific attention for its potential biological activities.

The obtained *C*OOs could be used as innovative and peculiar ingredients for pastry and baking application, thanks to their sweet note, in order to produce high quality foods. This possibility is due to the peculiar sensory properties of these oils, which are able to combine some nutritional qualities (antioxidant capacity, high phenol content, etc.) of the extra-virgin olive oil with the aromatic positive flavour due to the terpenes coming from the citrus peels.

## Figures and Tables

**Table 1:** Complete compositions of the essential oils of the peels of the lemon (*Citrus limon* (L.) Osbeck), both manually squeezed and hydrodistilled.

Constituents	l.r.i. ^1^	Relative Abundance (%)	
		Peels EO	Squeezed Peels VF ^2^
α-Thujene	931	0.4 ± 0.0	- ^3^
α-Pinene	941	1.6 ± 0.0	2.3 ± 0.1
Sabinene	976	2.0 ± 0.0	2.5 ± 0.1
β-Pinene	982	10.6 ± 0.4	14.9 ± 0.3
Myrcene	993	1.6 ± 0.1	0.5 ± 0.7
Octanal	1001	0.2 ± 0.0	-
α-Terpinene	1018	0.3 ± 0.0	-
*p*-Cymene	1027	0.3 ± 0.0	-
Limonene	1032	50.2 ± 0.3	70.3 ± 0.6
(*E*)-β-Ocimene	1052	0.2 ± 0.0	-
γ-Terpinene	1062	9.6 ± 0.6	9.6 ± 0.3
*cis*-Sabinene hydrate	1070	0.2 ± 0.0	-
Terpinolene	1088	0.6 ± 0.0	-
Linalool	1101	1.0 ± 0.1	-
Nonanal	1102	0.5 ± 0.1	-
Camphor	1143	0.1 ± 0.1	-
Citronellal	1155	0.4 ± 0.0	-
*Iso*neral	1171	0.2 ± 0.0	-
4-Terpineol	1178	0.6 ± 0.1	-
Isogeranial	1184	0.2 ± 0.0	-
α-Terpineol	1191	2.0 ± 0.1	-
Nerol	1230	0.8 ± 0.1	-
Neral	1240	5.7 ± 0.1	-
Geraniol	1257	0.8 ± 0.3	-
Geranial	1271	7.1 ± 0.0	-
Neryl acetate	1366	0.6 ± 0.1	-
Geranyl acetate	1385	0.4 ± 0.1	-
β-Caryophyllene	1420	0.3 ± 0.0	-
*trans*-α-Bergamotene	1438	0.4 ± 0.0	-
Valencene	1492	0.2 ± 0.0	-
Bicyclogermacrene	1495	0.2 ± 0.0	-
β-Bisabolene	1509	0.7 ± 0.1	-
Valerianol	1656	0.1 ± 0.0	-
Monoterpene hydrocarbons		77.3 ± 1.1	100.0 ± 0.0
Oxygenated monoterpenes		0.7 ± 0.1	-
Sesquiterpene hydrocarbons		20.1 ± 0.9	-
Oxygenated sesquiterpenes		1.8 ± 0.1	-
Non-terpene derivatives		0.1 ± 0.0	-
Extraction yield (% *w*/*w*)		0.57	-
Total identified (%)		100.0 ± 0.0	100.0 ± 0.0

^1^ Linear retention indices on a DB5 column; ^2^ Volatile fraction obtained by manual squeezing of the peels in n-hexane HPLC grade; ^3^ Not detected.

**Table 2:** Complete compositions of the essential oils of the peels of the orange (*Citrus sinensis* (L.) Osbeck), both manually squeezed and hydrodistilled.

Constituents	l.r.i. ^1^	Relative Abundance (%)	
		Peels EO	Squeezed Peels VF ^2^
α-Pinene	941	0.6 ± 0	0.9 ± 0.0
Sabinene	976	2.0 ± 0.1	3.2 ± 0.0
Myrcene	993	2.2 ± 0.1	2.6 ± 0.0
Octanal	1001	2.0 ± 0.0	0.5 ± 0.0
Limonene	1032	85.7 ± 0.2	91.4 ± 0.1
(*E*)-β-Ocimene	1052	- ^3^	0.1 ± 0.0
*n*-Octanol	1071	0.3 ± 0.0	-
Linalool	1101	3.5 ± 0.2	0.5 ± 0.1
Nonanal	1102	-	0.2 ± 0.0
*trans*-Limonene oxide	1141	0.1 ± 0.0	-
Citronellal	1155	0.1 ± 0.0	-
4-Terpineol	1178	0.3 ± 0.0	-
α-Terpineol	1189	0.5 ± 0.0	-
Decanal	1204	0.6 ± 0.0	0.3 ± 0.0
Citronellol	1230	0.1 ± 0.0	-
Neral	1240	0.4 ± 0.0	-
Geranial	1271	0.6 ± 0.0	-
Valencene	1492	0.9 ± 0.1	0.5 ± 0.1
Valerianol	1656	0.1 ± 0.1	-
Monoterpene hydrocarbons		90.5 ± 0.1	98.1 ± 0.2
Oxygenated monoterpenes		5.6 ± 0.0	0.5 ± 0.1
Sesquiterpene hydrocarbons		0.9 ± 0.1	0.5 ± 0.1
Oxygenated sesquiterpenes		0.1 ± 0.1	-
Non-terpene derivatives		2.9 ± 0.1	0.9 ± 0.0
Extraction yield (% *w*/*w*)		0.35	-
Total identified (%)		100.0 ± 0.0	100.0 ± 0.0

^1^ Linear retention indices on a DB5 column; ^2^ Volatile fraction obtained by manual squeezing of the peels in n-hexane HPLC grade; ^3^ Not detected.

**Table 3:** Complete headspace compositions of the lemon olive oil (*Citrus limon* olive oil *Cl*OO) and orange olive oil (*Citrus sinensis* olive oil *Cs*OO) compared to the unflavoured extra-virgin olive oil (EVOO control).

Constituents	l.r.i. ^1^	Relative Abundance (%)			Aroma Contribution ^2^
		(EVOO control)	*Cl*OO	*Cs*OO	
*n*-Hexanal	802	2.7 ± 0.4	- ^3^	-	Green, fruity
(*E*)-2-Hexenal	856	82.7 ± 2.3	0.3 ± 0.1	0.1 ± 0.0	Sweet, fruity, fragrant
*p*-Xylene	870	1.5 ± 0.1	-	-	
1-Hexanol	871	-	-	0.2 ± 0.0	
o-Xylene	897	1.6 ± 0.2	-	-	
3-Ethyl-1,5-octadiene (isomer 1)	898	0.7 ± 0.0	-	-	
3-Ethyl-1,5-octadiene (isomer 2)	901	0.5 ± 0.0	-	-	
α-Thujene	931	-	0.7 ± 0.1	-	
α-Pinene	941	-	2.9 ± 0.3	1.2 ± 0.2	Pine-, turpentine-like
1-Ethyl-4-methylbenzene	965	0.3 ± 0.4	-	-	
Sabinene	976	-	2.6 ± 0.0	1.5 ± 0.2	
β-Pinene	982	-	13.6 ± 0.6	0.9 ± 0.1	Dry, woody, resinous
Myrcene	993	-	2.6 ± 0.1	3.0 ± 0.2	Sweet, balsamic
*n*-Octanal	1001	-	-	0.5 ± 0.1	Citrus, honey-like
α-Terpinene	1018	-	0.3 ± 0.0	-	
1,2,4-Trimethylbenzene	1025	0.2 ± 0.2	-	-	
Limonene	1032	1.0 ± 0.7	67.1 ± 0.4	91.3 ± 0.2	Pleasant, lemon-like
(*E*)-β-Ocimene	1052	2.0 ± 0.3	0.1 ± 0.0	-	Warm herbaceous
γ-Terpinene	1062	-	8.1 ± 0.0	0.4 ± 0.1	Citrus, woody, bitter
Terpinolene	1088	-	0.5 ± 0.0	-	Citrus, pine-like
Linalool	1101	-	0.1 ± 0.0	0.6 ± 0.4	Pleasant, floral
*n*-Nonanal	1102	0.5 ± 0.8	-	0.1 ± 0.1	Citrus, rose-like
(*E*)-4,8-Dimethylnona-1,3,7-Triene	1116	0.8 ± 0.1	-	-	
*n*-Decanal	1204	-	-	0.1 ± 0.1	
(*E*)-2-Dodecene	1205	0.4 ± 0.5	-	-	
Neral	1240	-	0.2 ± 0.0	-	
Geranial	1271	-	0.3 ± 0.0	-	
Cyclosativene	1368	0.2 ± 0.3	-	-	
α-Copaene	1376	2.3 ± 0.2	-	-	
Valencene	1492	1.3 ± 0.0	-	-	
(*E*,*E*)-α-Farnesene	1507	0.4 ± 0.5	-	-	
Liguloxide	1532	0.7 ± 0.1	-	-	
Monoterpene hydrocarbons		3.0 ± 0.4	99.1 ± 0.1	98.3 ± 0.8	
Oxygenated monoterpenes		-	0.6 ± 0.0	0.6 ± 0.4	
Sesquiterpene hydrocarbons		4.2 ± 0.5	-	-	
Oxygenated sesquiterpenes		0.7 ± 0.1	-	-	
Non-terpene derivatives		92.1 ± 0.1	0.3 ± 0.0	1.1 ± 0.4	
Total identified (%)		99.9 ± 0.1	100.0 ± 0.0	100.0 ± 0.0	

^1^ Linear retention indices on a DB5 column; ^2^ Aromatic note of the compound [13] ^3^ Not detected.

**Table 4:** Chemical characterisation of control extra-virgin olive oil (Control EVOO), lemon olive oil (*Citrus limon* olive oil *Cl*OO), orange olive oil (*Citrus sinensis* olive oil *Cs*OO) and legal limits for extra-virgin olive oil according to the regulation EEC/2568/91 and later modifications and integrations [14].

	Reference Extra-Virgin Olive Oil (EEC Reg/2568/91 l.m.i.)	Control EVOO	*Cl*OO	*Cs*OO
Free Fatty Acidity (g oleic acid/kg oil)	≤0.80	0.18 ^a^	0.18 ^a^	0.18 ^a^
Peroxide Value (meq O_2_/kg oil)	≤20.00	5.00 ^a^	5.10 ^a^	5.00 ^a^
K_232_	≤2.50	1.48 ^a^	1.60 ^a^	1.52 ^a^
K_270_	≤0.22	0.12 ^a^	0.13 ^a^	0.16 ^a^
ΔK	≤0.10	0.00 ^a^	0.00 ^a^	0.00 ^a^

Within the same row, parameters sharing the same letter do not have a significantly different mean value.

**Table 5:** Total phenolic content, intensity of bitterness, antioxidant capacity and total carotenoid of lemon olive oil (*Citrus limon* olive oil *Cl*OO), orange olive oil (*Citrus sinensis* olive oil *Cs*OO), control extra-virgin olive oil (Control EVOO).

	Control EVOO	*Cl*OO	*Cs*OO
Total Phenol Content (TPC) (ppm gallic acid)	398 ^a^ **	242 ^b^ **	219 ^c^ **
Intensity of Bitterness (IB)	5.38 ^a^ **	2.19 ^c^ **	2.29 ^b^ **
Antioxidant capacity (AC) (μmol TEAC/mL)	0.27 ^a^	0.11 ^b^	0.12 ^b^
Total Carotenoid (TC) (mg/kg lutein)	0.98 ^b^ ***	0.94 ^b^ ***	5.88 ^a^ ***

Within each row significant differences are indicated by different letters. ***: *p* < 0.001; **: *p* < 0.01

**Table 6:** Changes in the composition of flavonoids (µg/mg phenols) in lemon olive oil (*Citrus limon* olive oil *Cl*OO), orange olive oil (*Citrus sinensis* olive oil *Cs*OO) and Control extra-virgin olive oil (Control EVOO).

	EVOO Control	*Cl*OO	*Cs*OO
Luteolin-7-*O*-glucoside	1.10 ± 0.02 ^b^	2.92 ± 0.07 ^a^	1.13 ± 0.09 ^b^
Rutin	0.05 ± 0.00 ^b^	n.d.	n.d.
Quercetin-3-*O*-glucoside	0.11 ± 0.00 ^a^	n.d.	n.d.
Apigenin-7-*O*-glucoside	3.45 ± 0.04 ^a^	0.04 ± 0.00 ^b^	0.01 ± 0.00 ^b^
Quercitrin	4.30 ± 0.07 ^a^	2.13 ± 0.09 ^c^	2.72 ± 0.08 ^b^
Quercetin-3-*O*-glucuronide	2.22 ± 0.08 ^a^	0.18 ± 0.00 ^b^	0.07 ± 0.00 ^c^
Quercetin	1.23 ± 0.03 ^a^	0.01 ± 0.00 ^c^	0.08 ± 0.00 ^b^
Luteolin	18.88 ± 0.32 ^b^	22.72 ± 0.30 ^a^	18.14 ± 0.67 ^b^
Kaempferol	20.37 ± 0.69 ^a^	2.70 ± 0.13 ^b^	0.18 ± 0.03 ^c^
Naringenin	n.d.	10.53 ± 0.49 ^a^	6.11 ± 0.59 ^b^
Total	51.71 ± 0.37 ^a^	41.22 ± 0.63 ^b^	28.45 ± 1.46 ^c^

Within each row significant differences (at *p* ≤ 0.05) are indicated by different letters; n.d. not detectable.

**Table 7:** Changes in the composition of phenolic alcohols, aldehyde and acids (µg/mg phenols) in lemon olive oil (*Citrus limon* olive oil *Cl*OO), orange olive oil (*Citrus sinensis* olive oil *Cs*OO), control extra-virgin olive oil (Control EVOO). Each value represents mean ± standard deviation (*n* = 3).

	EVOO Control	*Cl*OO	*Cs*OO
PHENOLIC ALCOHOLS			
Hydroxytyrosol	0.99 ± 0.004 ^b^	1.39 ± 0.04 ^a^	1.47± 0.03 ^a^
Tyrosol	28.36 ± 3.30 ^b^	98.05 ± 8.36 ^a^	93.46 ± 0.94 ^a^
PHENOLIC ALDEHYDES			
Vanillin	0.98 ± 0.05 ^a^	1.01 ± 0.04 ^a^	0.99 ± 0.01 ^a^
PHENOLIC ACIDS			
Chlorogenic acid	n.d.	0.20 ± 0.01 ^a^	0.18 ± 0.01 ^a^
Vanillic acid	0.23 ± 0.01 ^c^	1.29 ± 0.04 ^a^	0.74 ± 0.02 ^b^
Caffeic acid	0.12 ± 0.003 ^a^	0.13 ± 0.01 ^a^	0.12 ± 0.00 ^a^
Syringic acid	0.06 ± 0.002 ^a^	0.08 ± 0.01 ^a^	0.03 ± 0.01 ^b^
p-coumaric acid	0.14 ± 0.004 ^c^	0.50 ± 0.02 ^a^	0.40 ± 0.01 ^b^
Ferulic acid	0.68 ± 0.02 ^b^	9.42 ± 1.38 ^a^	2.50 ± 0.10 ^b^
Rosmarinic acid	n.d.	0.43 ± 0.02 ^b^	0.59 ± 0.04 ^a^
TOTAL	31.56 ± 3.31 ^b^	112.50 ± 7.12 ^a^	100.50 ± 1.03 ^a^

Within each row significant differences (at *p* ≤ 0.05) are indicated by different letters.

**Table 8:** Olive fruit characterisation.

Ripeness Index (0:7) [38]	3.5 ± 0.2
Average Weight (g)	1.56 ± 0.02
Average Volume (cm^3^)	1.67 ± 0.1
Water Content (%)	50.31 ± 0.03
Dry Matter (%)	49.69 ± 0.03
Oil Content (% d.m.)	32.20 ± 0.04

## Abbreviations

EVOO: Extra-virgin olive oil; EO: Essential oil; *C*OO: *Citrus* Olive oil, *Cl*OO: *Citrus limon* olive oil; *Cs*OO: *Citrus sinensis* olive oil.
